# Identification of Potential Biomarkers Associated with Acute Myocardial Infarction by Weighted Gene Coexpression Network Analysis

**DOI:** 10.1155/2021/5553811

**Published:** 2021-08-27

**Authors:** Yan Wang, Xiangyang Zhang, Min Duan, Chenguang Zhang, Ke Wang, Lili Feng, Linlin Song, Sheng Wu, Xuyan Chen

**Affiliations:** Department of Emergency, Beijing Tsinghua Changgung Hospital, School of Clinical Medicine, Tsinghua University, No. 168 Litang Road, Changping District, Beijing 102218, China

## Abstract

**Background:**

In the general population, acute myocardial infarction (AMI) represents a significant cause of mortality. This study is aimed at identifying novel diagnostic biomarkers to aid in treating and diagnosing AMI.

**Methods:**

The Gene Expression Omnibus (GEO) database was explored to extract two microarray datasets, GSE66360 and GSE48060, which were subsequently merged into a single cohort. Both AMI and control samples were analyzed for differentially expressed genes (DEGs), which were subsequently subjected to weighed gene coexpression network analysis (WGCNA) to identify the most significant module. Gene Ontology (GO) and pathway analyses subsequently carried out the most significant gene modules along with construction of a protein-protein interaction network (PPI). Cytoscape plugin cytoHubba allowed for the prediction of the top 4 key genes according to the network maximal clique centrality (MCC) algorithm. The expression levels and diagnostic value of the four key genes were additionally verified in the GSE62646 dataset.

**Results:**

A WCGNA analysis revealed 878 DEGs which were clustered into 6 modules. The module with the most significance in AMI was colored blue. Subsequent GO and KEGG pathway enrichment analysis on blue module genes revealed that they were primarily enriched in the inflammation-related pathways. These findings, in combination with PPI and coexpression networks, resulted in the identification of the top four genes by cytoHubba, which included leukocyte immunoglobulin-like receptor B2 (LILRB2), toll-like receptor 2 (TLR2), neutrophil cytosolic factor 2 (NCF2), and S100A9. Among them, LILRB2, NCF2, and S100A9 were validated in the GSE62646 dataset.

**Conclusions:**

The results suggested that LILRB2, NCF2, and S100A9 could be potential gene biomarkers for AMI.

## 1. Introduction

Acute myocardial infarction (AMI) denotes the presence of acute myocardial injury detected by abnormal cardiac biomarkers in the setting of evidence of acute myocardial ischaemia [1] and carries high mortality [2]. The diagnosis of AMI is made using a combination of clinical acumen, serum biomarker tests, and electrocardiographic analysis. Commonly used AMI biomarkers used in the clinical setting are comprised of myoglobin, creatine kinase-MB (CK-MB), cardiac troponin I (cTnI), and cardiac troponin T (cTnT) [3, 4]. However, elevated levels of these biomarkers also can be detected in patients with heart failure, renal failure, and thyroid disease [5]. It is therefore important to discover more AMI-specific biomarkers.

The advancement of bioinformatics and microarray analyses has resulted in higher numbers of novel gene discovery. In previous studies by our group, we carried out microarray analysis on peripheral blood samples belonging to AMI patients [6] as well as in mouse myocardial tissue [7] to determine the presence differentially expressed genes (DEGs). However, intergene relationships were more fully explored. Weighted gene coexpression network analysis (WGCNA) is a simple analysis method that allows for the construction of gene expression networks through clustering of highly correlated genes into modules [8]. This method enables the visualization of genes which are most representative of AMI. Central elements of these biological networks are more likely to represent essential genes which exhibit more important functions. The Cytoscape plugin cytoHubba provides a user-friendly interface to explore important nodes in biological networks [9], allowing for the identification of critical key genes.

This investigation involves two microarray datasets of AMI (GSE66360 and GSE48060) which were downloaded from GEO. A single cohort was formed by merging the two datasets. We systematically analyzed clusters of differentially expressed genes (DEG) which possessed similar expression patterns with WGCNA. The blue module was found to be strongly associated to AMI. Four key genes were identified from further analysis of the blue module. Three of these appeared to be correlated well in the GSE62646 correlation dataset and may represent potential candidate biomarkers of AMI.

## 2. Materials and Methods

### 2.1. Microarray Data Sources

The Gene Expression Omnibus (GEO) database (http://www.ncbi.nlm.nih.gov/geo/) was explored, and two independent AMI gene expression datasets were selected for this study (GSE66360 and GSE48060). These two datasets were derived from the chip-based platform GPL570 [HG-U133_Plus_2] Affymetrix Human Genome U133 Plus 2.0 Array. GSE66360 consisted of 99 circulating endothelial cell samples from 49 AMI patients and 50 patients without AMI, and GSE48060 comprised 52 and 21 serum samples from AMI and without AMI patients, respectively. No ethical approval was required given the lack of human or animal subjects in this study.

### 2.2. DEG Identification

The R software (version 3.6.1) and Bioconductor Packages (version 3.10) were used for data mining and statistical analyses. The “ComBat” tool from the R-package “sva” was used to merge the 2 datasets into a metadata cohort, removing batch effects. The raw data were first subjected to background correction and quantile normalization using the Affy package of R/Bioconductor [10]. We screened for significant DEGs using the Limma (linear models for microarray data) tool [11]. An adjusted false discovery rate *P* < 0.01 and fold change (FC) > 1.5 were set as the threshold for identifying significant DEGs. The R-package “ggplot2” and “pheatmap” were used to visualize the volcano plot and heatmap.

### 2.3. Weighted Gene Coexpression Network Analysis and Hub Gene Identification

A weighted gene coexpression network (WGCNA) algorithm was performed for the analysis of the coexpression network as well as to determine hub genes [12]. In brief, the WGCNA R package (version 1.68) allowed for WGCNA analysis. The soft threshold power was calculated using the function pickSoftThreshold of the WGCNA package. In this function, soft threshold was chosen as 8 for the correlation matrix. Based on the matrix, a topological overlap matrix (TOM) was used to measure similarity. Genes were then hierarchically clustered and visualized in a dendrogram according to the dissimilarity TOM (1-TOM).

Each first principal component of each gene module was determined as the module eigengenes (MEs). The ME expression was then taken to represent all genes in each module. We then sought for correlations between clinical features and MEs in order to determine AMI-associated modules [13]. Gene significance (GS), defined as the absolute correlation between the gene and the trait, was used to identify the associations between each gene and AMI. The module membership (MM) was determined to be the degree of correlation between MEs and gene expression profiles. Correlations between MM and GS were analyzed to determine modules of interest. Hub genes were those with a MM > 0.8 and GS > 0.2.

### 2.4. Functional and Pathway Enrichment Analysis of Hub Genes

The R package clusterProfiler (version 3.14.3) was used for gene ontology (GO) enrichment analysis [14]. Bioconductor provided the reference database located at http://org.Hs.eg.db 3.10.0 with options being fun = ^“^enrichGO,” pAdjustMethod = ^“^BH,” pvalueCutoff = 0.01, and qvalueCutoff = 0.05.

Signaling pathways of hub genes were investigated with reference to the Kyoto Encyclopedia of Genes and Genomes (KEGG) [15], which collects pathway-related information regarding molecule networks. The R software clusterProfiler was used to carry out KEGG pathway enrichment analyses [14]. Statistical significance was granted when *P* value < 0.05.

### 2.5. PPI Network Construction and Key Gene Screening

We used the online database Search Tool for the Retrieval of Interacting Genes (STRING; http://string-db.org) for prediction of protein-protein interaction (PPI) networks [16]. PPIs located in AMI-associated module hub genes were screened and selected based on a confidence score > 0.4. The networks were then imaged using the Cytoscape software (version 3.7.2). A plugin of Cytoscape, cytoHubba, enabled us to predict the top 4 crucial genes according to the maximal clique centrality (MCC) algorithm [9].

### 2.6. Validation of Key Genes

GSE62646, comprising of serum samples from 28 ST-segment elevation myocardial infarction (STEMI) patients and 14 patients with stable coronary artery disease (CAD) as controls, was extracted from the GEO database and utilized as a validation dataset. According to the GPL6244 [HuGene-1_0-st] Affymetrix Human Gene 1.0 ST Array annotation platform, the probe annotation was performed. Identification of DEGs was performed utilizing the Limma package. Unpaired Student's *t*-test from the R software was used to contrast intergroup gene expression variances. The diagnostic value of the 4 selected hub genes was assessed using a receiver operating characteristic (ROC) curve created using the “pROC” package in R [17], and MedCalc (MedCalc 19.4.1 version, MedCalc Inc., Mariakerke, Belgium) and was based on the GSE62646 dataset.

## 3. Results

### 3.1. Identification of DEGs

**Figure 1:**
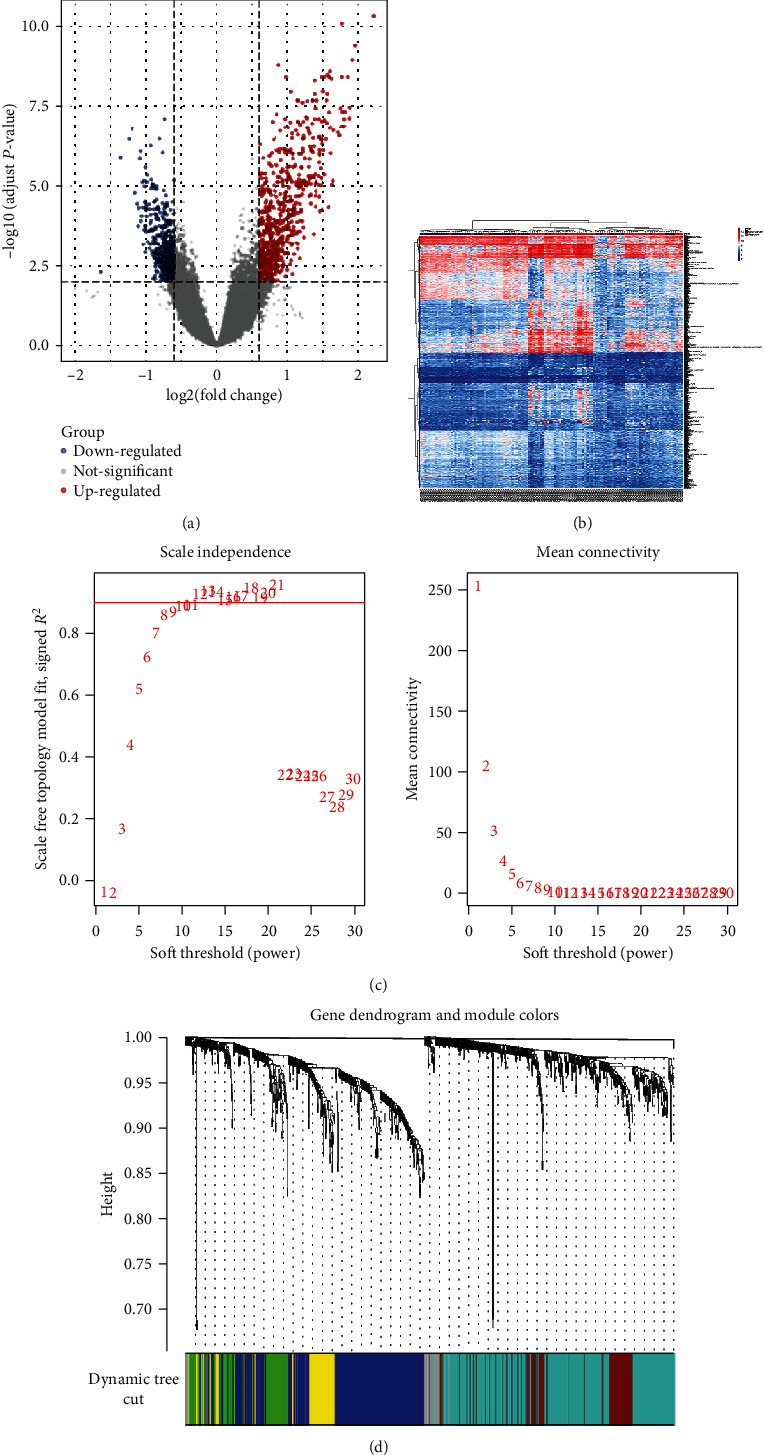
WGCNA of DEGs in AMI. (a) Volcano plots of DEGs. (b) AMI DEGs visualized using a heatmap. (c) Soft thresholding power analysis allowed for provision of scale-free fit index of network topology. (d) Coexpression clusters were conducted with hierarchical cluster analysis with corresponding color assignments encoded using WGCNA.

878 DEGs which conformed to the selection criteria were identified, and 450 upregulated DEGs and 428 downregulated DEGs were included. These are visualized in volcano plots in which each dot represents a gene (Figure 1(a)).  Figure 1(b) represents a heatmap of the expression levels of these DEGs.

### 3.2. Construction of Weighted Coexpression Networks

AMI hub genes were assessed by WGCNA studies on the coexpression network of the 878 DEGs. The power value is the most important variable that may impact the average degree of connectivity and independence of the coexpressed modules. Various soft threshold powers were applied on the screened network topology, with *β* = 8 selected for later analysis (Figure 1(c)). WGCNA was used to construct a gene coexpression network based on the hierarchical clustering of the predetermined dissimilarities. Six modules were then obtained (Figure 1(d)).

### 3.3. Identifying Genes in Blue Module Associated with AMI

**Figure 2:**
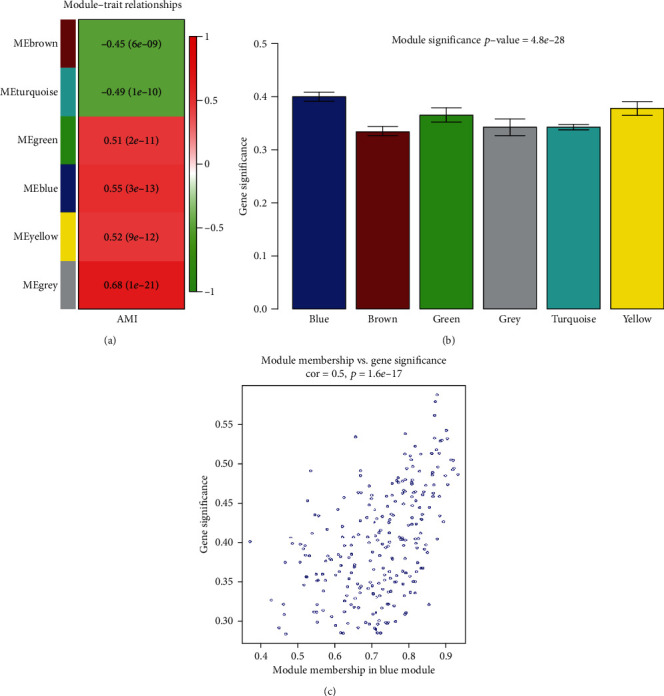
Significant modules associated with AMI. (a) Correlation of module eigengenes with AMI. (b) Bar plot of average gene significance of each AMI-associated gene. (c) Correlation between gene significance and MEblue membership.

All gene modules were also studied in association with clinical features, resulting in six identified modules which demonstrated evidence of association with *P* < 0.05 (Figure 2(a)). Genes that were not able to be clustered are represented with the grey module. Those belonging to the blue module were significantly positively correlated with AMI (*r* = 0.55, *p* = 3*e* − 13). The module significance of the blue module was higher than that of any other, suggesting its stronger connection with AMI (Figure 2(b)). Genes included in the blue module were also significantly associated with gene significance and are plotted in Figure 2(c).

### 3.4. GO Function and KEGG Pathway Annotation of Module Hub Genes

**Figure 3:**
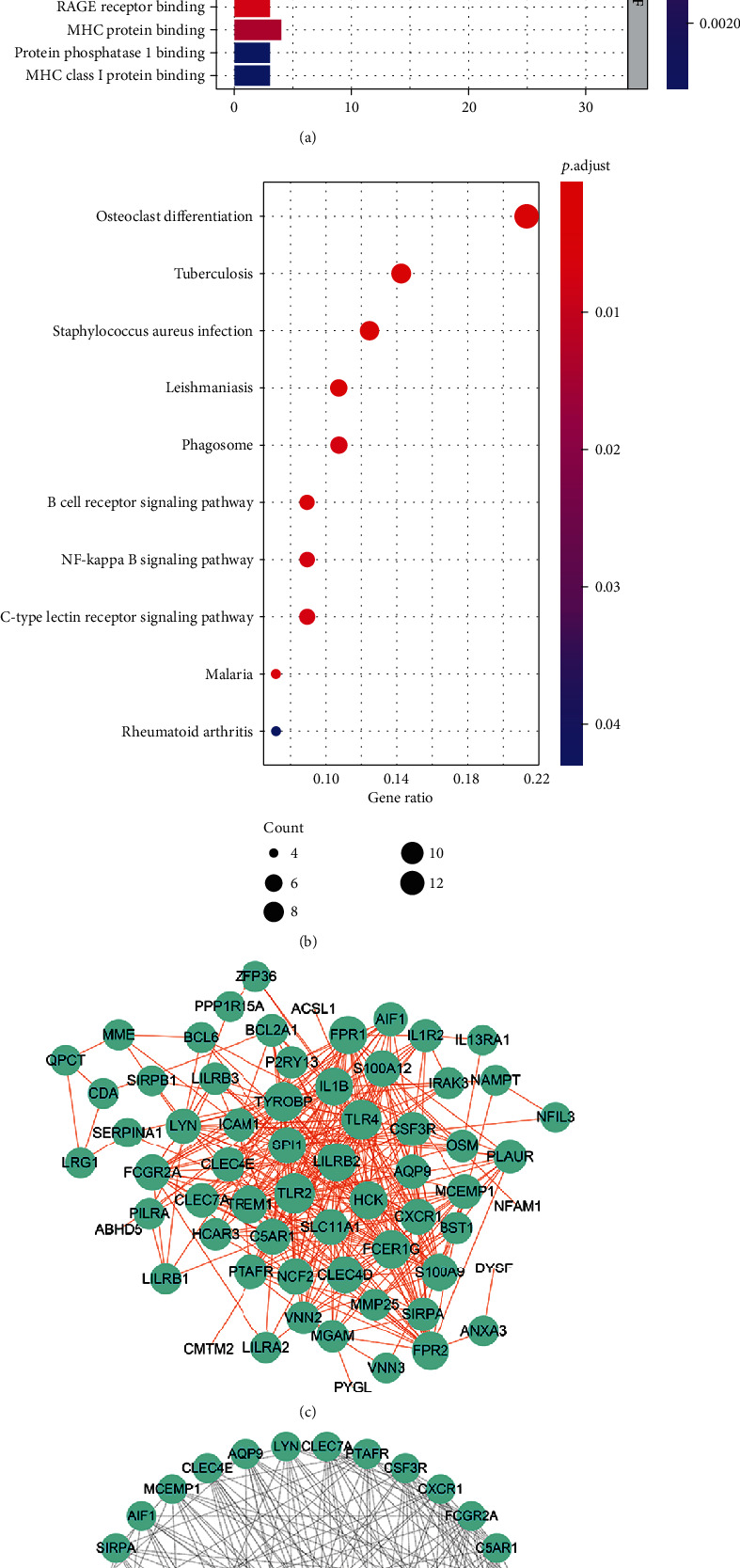
Function and pathway enrichment analysis and key gene cluster. (a) The GO function enrichment of DEGs in the blue module. (b) KEGG pathway enrichment of the DEGs in the blue module. (c) PPI network of genes in blue module. (d) Top 4 key genes explored by cytoHubba. GO: Gene Ontology; DEGs: differentially expressed genes; KEGG: Kyoto Encyclopedia of Genes and Genomes; PPI: protein-protein interaction.

GO function and KEGG pathway enrichment analyses were performed to assess the function of 255 genes in the blue module. 479 enriched GO terms were in biological process (BP), 22 enriched GO terms were in cellular component (CC), and 19 enriched GO terms were in molecular function (MF). The top 8 BP, CC, and MF terms are shown in Figure 3(a). 11 significantly enriched KEGG pathways were identified in this module. The most significant KEGG pathways included osteoclast differentiation, tuberculosis, and staphylococcus aureus infection. The top 10 KEGG pathways are shown in Figure 3(b).

### 3.5. PPI Network Construction and Key Genes Identification

The 255 genes in the blue module were used to construct a PPI network, which was composed of 74 nodes and 361 edges, extracted from the STRING database and imaged with the Cytoscape software (Figure 3(c)). The top 4 key genes were identified by the MCC method utilizing the cytoHubba plug-in of Cytoscape, including leukocyte immunoglobulin-like receptor B2 (LILRB2), toll-like receptor 2 (TLR2), neutrophil cytosolic factor 2 (NCF2), and S100A9 (Figure 3(d)).

### 3.6. Validation of Key Genes Using Other Datasets

**Figure 4:**
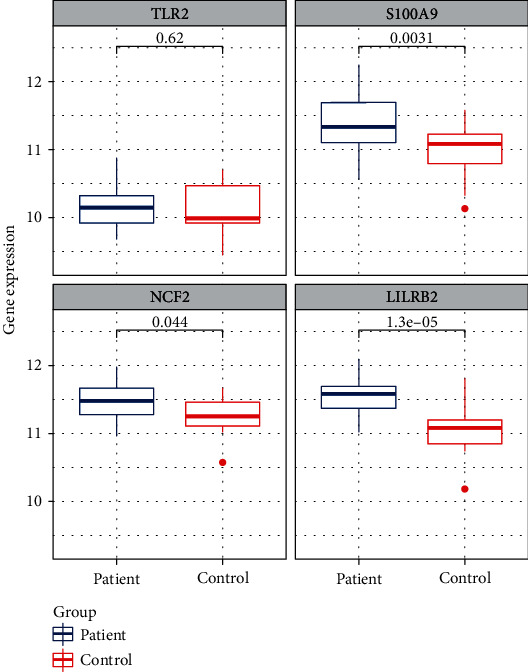
Contrast of the expression change of TLR2, S100A9, NCF2, and LILRB2 in the GSE62646 dataset. The box plots represent the results from the AMI group and control group. *P* values are shown above each box plot.

Data validation was performed using the GSE62646 dataset. We analyzed the expression levels of LILRB2, TLR2, NCF2, and S100A9 in AMI samples (blood samples on the first day of myocardial infarction) and stable coronary artery disease (CAD) controls (Figure 4). The expressions of LILRB2, NCF2, and S100A9 were noted to be significantly changed between the AMI and CAD groups while that of TLR2 was not remarkably different.

**Figure 5:**
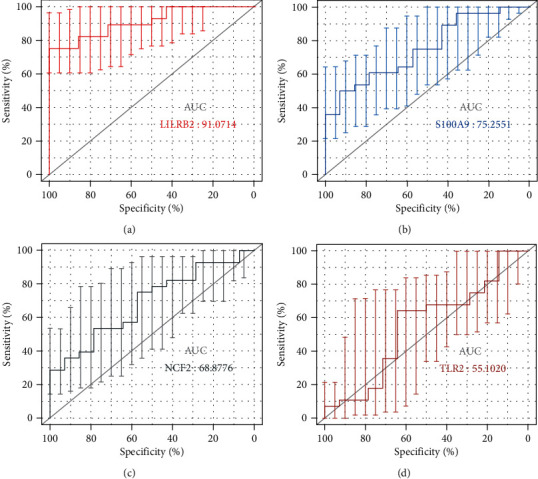
ROC curves of the 4 key genes. (a–d) ROC curves for LILRB2, S100A9, NCF2, and TLR2, respectively. The error bars represent the standard deviation of measurements for 28 STEMI patients and 14 patients with stable CAD as controls (*n* = 42).

These three genes also demonstrated powerful discrimination ability in the GSE62646 dataset with an AUC of 0.911 (95% CI: 0.827–0.995) in LILRB2, AUC of 0.753 (95% CI: 0.604–0.901) in S100A9, AUC of 0.689 (95% CI: 0.525–0.853) in NCF2, and AUC of 0.551 (95% CI: 0.356–0.746) in TLR2 (Figure 5). Among them, the combined diagnostic ability of LILRB2 demonstrated an AUC over 0.9, highlighting its strong diagnostic ability. TLR2 on the other hand demonstrated a poor diagnostic performance.

Both sensitivity and positive predictive values of LILRB2 are very high; thus, LILRB2 will allow for the early identification of AMI. The optimized cut-point value of LILRB2 is 11.38, with a specificity of 100%. LILRB2 over 11.38 may predict early AMI and facilitate the evaluation of early intervention trials (Table 1).

## 4. Discussions

AMI is the primary cause of global morbidity and mortality. The occurrence and development of AMI are a multifactorial process that has largely been identified; however, there is a lack of highly specific diagnostic and therapeutic biomarkers for this disease. Hence, it is important to explore genes associated with the mechanisms of AMI. Our investigation identifies critical genes which correlate strongly with AMI with the help of bioinformatics analysis of available microarray data.

In the study, 878 DEGs comprising of 450 upregulated and 428 downregulated genes were discerned from the GSE66360 and GSE48060 datasets. Gene correlation at the RNA level is able to be explored using WGCNA-based gene coexpression network analyses. Based on WGCNA, we found that AMI-specific genes may be represented by the blue module. Subsequent GO functional enrichment analysis clarified that DEGs were primarily enriched in the following functional categories, including neutrophil activation and degranulation involved in immune response. It is widely accepted that neutrophil-mediated cardiovascular occlusion is an important phenomenon in AMI [18]. Strong, short bursts of neutrophil activation have been documented to occur in the early phases of an AMI and have been linked to trigger thrombotic vessel occlusion [19]. KEGG pathway enrichment analysis revealed DEGs to be mainly enriched in the inflammation-related pathways, such as Staphylococcus aureus infection, phagosome, NF-kappa B signaling pathway, and C-type lectin receptor signaling pathway. The inflammatory cascade is important in the occurrence of AMI [20]. The NF-kappa B has been repeatedly emphasized to play a central role in inflammation-mediated cardiac remodelling post-AMI [21, 22]. These results offer a glimpse in the biological function and associated gene pathways in AMI progression.

Genes belonging to the blue module were then utilized in PPI network construction using the STRING database. The top four hub genes were identified by cytoHubba, which included LILRB2, TLR2, NCF2, and S100A9. The GSE62646 dataset was used to validate the relationship of these 4 key genes in AMI. With the exception of TLR2, the expression levels of the other three genes were markedly different in AMI samples in contrast to control samples. ROC curves were constructed in order to further discern the diagnostic utility of these biomarkers. As expected, only TLR2 demonstrated inadequate diagnostic utility. Both S100A9 and NCF2 were of acceptable diagnostic values. Moreover, the AUC of LILRB2 was more than 0.90, which indicated a good diagnostic ability. Thus, LILRB2, S100A9, and NCF2 may be critical in AMI progression.

S100A9 is rapidly released in response to inflammatory stimuli and acts as a potent activator of the innate immune response in conditions which demonstrate immune and inflammatory components, such as AMI [23]. S100A9 has been identified as a potential therapeutic target in treating AMI [24]. It has recently been shown that S100A9 represents a gene that is highly upregulated in the myocardium in the immediate postischemic period, further supporting its role as an important first responder to ischemic injury [25, 26]. Moreover, high levels of S100A9 in AMI patients during the first 24 hours post-MI appeared to be related in higher risks of major adverse cardiovascular events and heart failure [25, 27]. Similarly, our study found S100A9 to be upregulated in AMI patients of both the GSE66360 and GSE48060 datasets.

LILRB2 is a member of the leukocyte immunoglobulin-like receptor family, which can negatively regulate immune cell activation by acting on intracellular immunoreceptor tyrosine-based inhibitory motifs (ITIMs) [28]. Limited evidence hints towards a positive association between LILRB2 expression and AMI progression [29, 30]. In our study, LILRB2 was primarily enriched in the signaling pathway related to T cell activation, a finding that is in accordance with previously documented functions of LILRB2 [31]. T cells are considered to be the primary immune competent cells that modulate atherosclerotic plaque formation [32]. T cell-associated cytokine imbalance has been confirmed to be related to AMI and may predict outcomes in those with ischemic heart disease outcomes [33, 34]. However, the role of LILRB2 in AMI requires further confirmation.

NCF2 is part of the leukocyte NADPH oxidase complex responsible for producing superoxides. Several autoimmune conditions such as systemic lupus erythematosus (SLE) and Crohn's colitis have been found to harbor mutations in this gene [35, 36]. A report suggests that NCF2 may mediate changes in blood pressure along with cerebral strokes related to unstable atherosclerotic plaques [37, 38]. However, no research has linked NCF2 to AMI. It has been well established that high levels of reactive oxygen species (ROS) are deleterious and can result in myocardial infarction [39, 40]. NADPH oxidases are primary nonmitochondrial sources of ROS. These membrane-associated multiprotein complexes, including NFC2, are essential and crucial components. NFC2 may be a pathogenic factor and potential target in diagnosing and treating AMI.

Despite the discovery of 3 novel AMI-associated genes, our study is limited by its retrospective design which inevitably contained some degree of incomplete clinical information. The reproducibility of our results should also be validated across additional datasets to enhance its robustness.

## 5. Conclusions

In conclusion, this study identified 4 key genes through WGCNA analysis which may contribute towards AMI development. The function of three of these genes, S100A9, LILRB, and NCF2, performed well in the validation dataset and may act as potential biomarkers for AMI.

## Figures and Tables

**Table 1:** Diagnostic accuracy of 4 key genes.

Key genes	AUC (95% CI)	Cut-point value	Specificity (%) (95% CI)	Sensitivity (%) (95% CI)	PPV (%) (95% CI)	NPV (%) (95% CI)
TLR2	0.551 (0.39-0.71)	10.01	64.29 (0.35-0.87)	64.29 (0.44-0.81)	78.3 (0.63-0.89)	47.4 (0.32-0.63)
S100A9	0.753 (0.60-0.87)	11.31	92.86 (0.66-1.00)	50 (0.31-0.69)	93.3 (0.67-0.99)	48.1 (0.38-0.58)
NCF2	0.689 (0.53-0.82)	11.27	57.14 (0.28-0.82)	75.00 (0.55-0.89)	77.8 (0.65-0.87)	53.3 (0.34-0.72)
LILRB2	0.911 (0.78-0.98)	11.38	100 (0.77-1.00)	75.00 (0.55-0.89)	100 (-)	66.7 (0.51-0.79)

## Data Availability

The data used to support the findings of this study are available from the corresponding author upon request.
